# Heat-killed *Lacticaseibacillus paracasei* GMNL-653 ameliorates human scalp health by regulating scalp microbiome

**DOI:** 10.1186/s12866-023-02870-5

**Published:** 2023-04-29

**Authors:** Wen-Hua Tsai, Yi-Ting Fang, Tsuei-Yin Huang, Ying-Ju Chiang, Ching-Gong Lin, Wen-Wei Chang

**Affiliations:** 1 Research and Development Department, GenMont Biotech Incorporation, Tainan, Taiwan; 2 Bachelor Program in Cosmeceutical and Biotech Industry, Department of Cosmetic Science, Chia Nan University of Pharmacy & Science, Tainan, Taiwan; 3 Department of Biomedical Sciences, Chung Shan Medical University, No. 110, Section 1, Chien-Kuo N. Rd, Taichung City, 402306 Taiwan; 4 Department of Medical Research, Chung Shan Medical University Hospital, Taichung, Taiwan

**Keywords:** Heat-killed probiotics, *Lacticaseibacillus paracasei*, Shampoo, Scalp health care, Scalp microbiome

## Abstract

**Background:**

The equilibrium of the scalp microbiome is important for maintaining healthy scalp conditions, including sebum secretion, dandruff, and hair growth. Many different strategies to improve scalp health have been reported; however, the effect of postbiotics, such as heat-killed probiotics, on scalp health remains unclear. We examined the beneficial effects of heat-killed probiotics consisting of *Lacticaseibacillus paracasei*, GMNL-653, on scalp health.

**Results:**

Heat-killed GMNL-653 could co-aggregate with scalp commensal fungi, *Malassezia furfur*, in vitro, and the GMNL-653-derived lipoteichoic acid inhibited the biofilm formation of *M. furfur* on Hs68 fibroblast cells. The mRNA of hair follicle growth factors, including insulin-like growth factor-1 receptor (IGF-1R), vascular endothelial growth factor, IGF-1, and keratinocyte growth factor was up-regulated in skin-related human cell lines Hs68 and HaCaT after treatment with heat-killed GMNL-653. For clinical observations, we recruited 22 volunteer participants to use the shampoo containing the heat-killed GMNL-653 for 5 months and subsequently measured their scalp conditions, including sebum secretion, dandruff formation, and hair growth. We applied polymerase chain reaction (PCR) to detect the scalp microbiota of *M. restricta*, *M. globosa*, *Cutibacterium acnes*, and *Staphylococcus epidermidis*. A decrease in dandruff and oil secretion and an increase in hair growth in the human scalp were observed after the use of heat-killed GMNL-653-containing shampoo. The increased abundance of *M. globosa* and the decreased abundance of *M. restricta* and *C. acnes* were also observed. We further found that accumulated *L. paracasei* abundance was positively correlated with *M. globosa* abundance and negatively correlated with *C. acnes* abundance. *S. epidermidis* and *C. acnes* abundance was negatively correlated with *M. globosa* abundance and positively correlated with *M. restricta*. Meanwhile, *M. globosa* and *M. restricta* abundances were negatively associated with each other. *C. acnes* and *S. epidermidis* abundances were statistically positively correlated with sebum secretion and dandruff, respectively, in our shampoo clinical trial.

**Conclusion:**

Our study provides a new strategy for human scalp health care using the heat-killed probiotics GMNL-653-containing shampoo. The mechanism may be correlated with the microbiota shift.

**Supplementary Information:**

The online version contains supplementary material available at 10.1186/s12866-023-02870-5.

## Introduction

Human scalp is covered with high density of hair growing from hair follicles with connection to sebaceous glands, creating a lipid-rich hydrophobic niche that harbors a variety of microorganisms [1]. Cutaneous microorganism of fungi and bacteria are strongly associated with scalp disorders and diseases, including dandruff, seborrheic dermatitis (SD), scalp psoriasis, folliculitis decalvans, and alopecia [2]. Fungal *Malassezia* is one of the etiologic factors in the progression of dandruff [3] and the source of oxidative damage that leads to premature hair loss [4] and plays a role in the pathogenesis of alopecia [5]. Bacteria *Cutibacterium* and *Staphylococcus* also contribute to scalp inflammation, hair-follicle associated disorders, and hair diseases [6]. Metagenomic analysis showed that *Malassezia*, *Cutibacterium*, and *Staphylococcus* are the main fungal and core bacterial genera found on healthy and dandruff scalps, and the species of *M. restricta*, *M. globosa*, *C. acnes*, and *S. epidermidis* are further identified [7–9]. Furthermore *M. restricta* and *M. globosa* display contrary roles in scalp health, and *C. acnes* and *S. epidermidis* are reciprocally inhibited bacteria on the scalp [7, 8]. The role of scalp microbiota diversity in causing scalp disorders and diseases has been highlighted and investigated. For example, the diversity of microbial composition in hair follicle contributes to the pro-inflammatory environment in chronic inflammatory scalp disease [10]. High bacterial diversity was detected in the lesions of patients with psoriasis and SD compared with that in the healthy controls [11]. The intraspecific diversity of *Malassezia* from the scalp microbiota was found in patients with SD [12]. Scalp microorganisms can also affect the scalp situation through microbial cell–host interaction. The fungal microbiome is majorly implicated in cell–host adhesion in dandruff scalp, and the bacterial microbiome is related to the synthesis of biotin, vitamins, and other nutrients in healthy scalp [8].

Several bio-oils and plant extracts, including anti-dandruff and hair growth stimulating agents, are used as scalp-related products to maintain scalp health and hygiene [13–15]. Recent evidence has shown the beneficial effects of probiotics for skin health, such as maintaining barrier integrity, tissue homeostasis, and microbiome balance and inhibiting pathogen growth, biofilm formation, and local inflammation [1, 16, 17]. The beneficial effects of probiotic intake on modulating cell cycles in hair follicles and promoting hair growth have also been reported [18, 19]. Live probiotics and the compounds from bacteria or their metabolites play important role in skin care. For example, the fermentation metabolites of commensal *S. lugdunensis* could suppress fungi overgrowth, providing novel therapeutics for *C. parapsilosis*-associated infection in human dandruff [20]. The glycolipopeptide of *Lactiplantibacillus pentosus* and *Lacticaseibacillus paracasei* has anti-adhesion and anti-microbial activity against pathogen in the skin microflora [21]. Our previous studies demonstrated that heat-killed *Lactobacilli* preparations from *L. plantarum* GMNL-6 and *L. paracasei* GMNL-653 promote skin wound healing and preventing excessive fibrosis by suppressing TGF-β/pSmad signaling [22]. Lipoteichoic acid, the major cell wall component of GMNL-6, has a beneficial effect on skin care [23]. Heat-killed GMNL-653 also exhibits anti-osteoporotic effects by regulating the gut microbiota dysbiosis [24]. Given the safety concern of live probiotics in immunosuppressive populations, the preparations of inanimate microorganisms or their components have potential benefits and l applications for skin care [25]. In the present study, we investigated the potential role of heat-killed *L. paracasei* GMNL-653 in human scalp health using in vitro cell lines and recruiting human participants.

## Results

### Heat-killed GMNL-653 has beneficial effects on co-aggregation with *M. furfur* and inhibitory effects on the biofilm formation of *M. furfur*

**Fig. 1:**
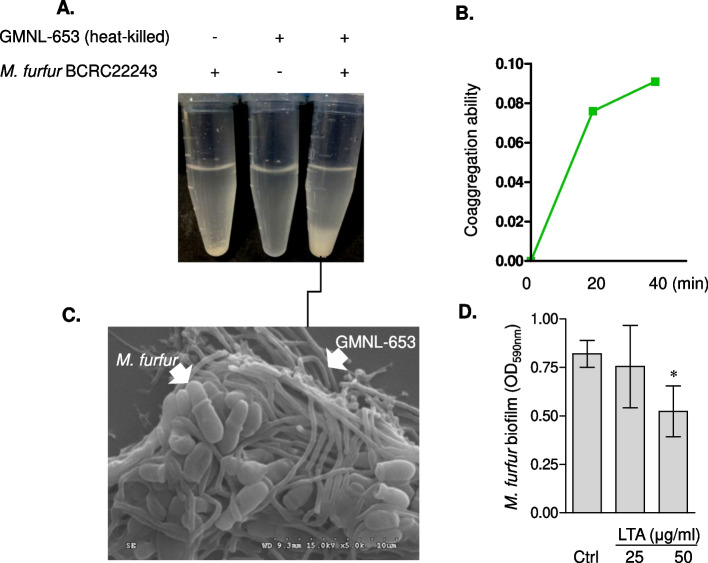
Heat-killed GMNL-653 causes co-aggregation with *M. furfur* and inhibits its adhesion to Hs86 cells. **A**,** B** Heat-killed GMNL-653 (2 × 10^9^ cells/ml), live *M. furfur* (2 × 10^9^ cfu/ml), or mixtures of heat-killed GMNL-653 and live M. furfur with the ratio of 1:1 were added into tubes and stand at room temperature for 40 min to observe the formation of precipitates (**A**). Liquids of the suspended area from the tubes of M. *furfur* alone, GMNL-653 alone, and M. *furfur*-GMNL mixture were collected after mixing for 0, 20, and 40 min. The liquid was detected by the absorbance at a wavelength of 590 nm (**B**). The aggregation ability was quantified by the formula described in the Materials and Methods section. **C** The aggregation of GMNL-653 and *M. furfur* from (**A**) was visualized by SEM. **D** *M. furfur* were seeded into wells of a 96-well-plate with or without adding 25, 50 µg/ml GMNL-653 derived LTA for 24 h. The biofilm was visualized after staining with 0.1% crystal violet, dissolved with DMSO, and quantified by the absorbance at a wavelength of 590 nm. *, *p* < 0.05

We aimed to clarify whether heat-killed probiotics GMNL-653 has effects on scalp pathological microbes in vitro. First, we evaluated the ability of heat-killed GMNL-653 to co-aggregate with fungus *M. furfur*, a common pathological inhabitant on human scalp. We found that when mixed in a test tube, heat-killed GMNL-653 aggregated with *M. furfur* (Fig. 1A) and increased following times (Fig. 1B). The co-aggregation of heat-killed GMNL-653 and *M. furfur* was also confirmed by SEM (Fig. 1C). Furthermore, we evaluated whether GMNL-653-derived LTA, the major cell wall component, influence the biofilm formation of *M. furfur* on human scalp. The results showed that GMNL-653-derived LTA inhibited the biofilm formation of *M. furfur on* Hs86 cells in a dose-dependent manner (Fig. 1D). Owing to its capabilities of co-aggregating with *M. furfur* and reducing the biofilm formation of *M. furfur*, heat-killed GMNL-653 potentially has beneficial effects on scalp health.

### Heat-killed GMNL-653 up-regulates the expression of growth factors in skin cell lines

**Fig. 2:**
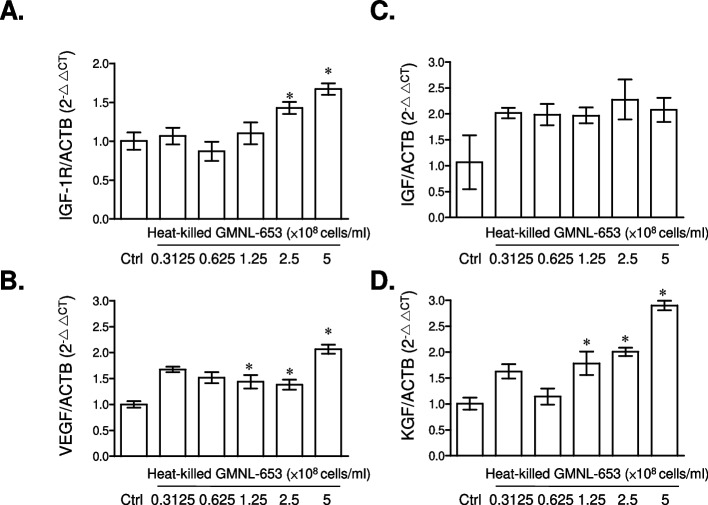
Treatment of heat-killed GMNL-653 in Hs68 fibroblasts increases the mRNA expressions of growth factors. 1.5 × 10^5^ cells/well of human fibroblast Hs68 cells were seeded in wells of 6-well-plate for attachment and then cultured with serum free medium for 24 h. Cells were treated with indicated concentration of heat-killed GMNL-653 for 24 h. The mRNA expression of IGF-1R (**A**), VEGF (**B**), IGF-1 (**C**), and KGF (**D**) were determined by quantified RT-PCR (*n* = 5). Data were presented as the relative fold changes (mean ± SEM) in compared to non-GMNL-653 treatment control (Ctrl) after normalization with the house-keeping of β-actin. *p < 0.05

**Fig. 3:**
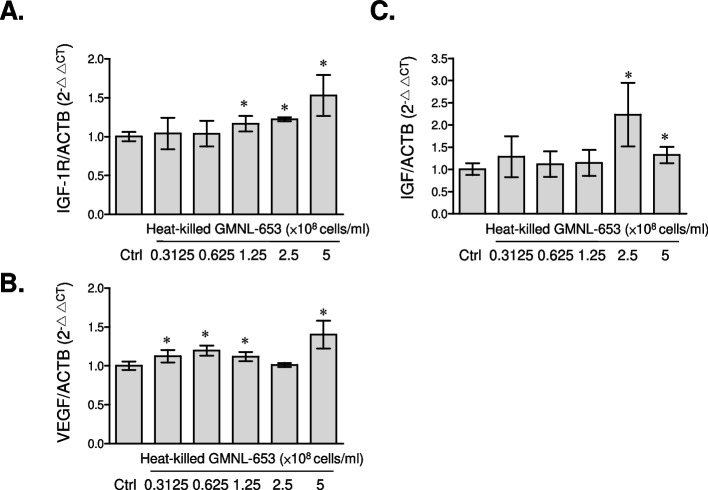
Heat-killed GMNL-653 increases the mRNA expressions of growth factors in HaCaT keratinocytes. Human epidermal keratinocyte HaCaT cells were seeded in 6-well-plate as a cell density of 3 × 10^5^ cells/well and cultured with serum free medium after attachment for 24 h. Cells were treated with the indicated concentrations of heat-killed GMNL-653 for further 24 h. The mRNA expressions of IGF-1R (**A**), VEGF (**B**), and IGF-1 (**C**) were determined by qRT-PCR. Data were presented as the relative fold changes (mean ± SEM) in compared to non-GMNL-653 treatment control (Ctrl) after normalization with the house-keeping of β-actin. *p < 0.05

Next, we examined the effect of heat-killed GMNL-653 on the expression of growth factors in human skin cells. The mRNA expression of growth factor genes in Hs68 cells and HaCaT cells, human immortal keratinocytes, was determined. The results showed that the mRNA expression of IGF-1R (Fig. 2A), VEGF (Fig. 2B), and KGF (Fig. 2D) was up-regulated in Hs68 cells after treatment with heat-killed GMNL-653 in a dose-dependent manner. IGF expression was also up-regulated, though the difference was not statistically significant (Fig. 2C). We also observed that heat-killed GMNL-653 up-regulated the mRNA expression of IGF-1R (Fig. 3A), VEGF (Fig. 3B), and IGF (Fig. 3C) in HaCaT keratinocytes. Basing on the up-regulation of these growth factors, we suggested that heat-killed GMNL-653 could improve scalp hair growth.

### Beneficial effects of heat-killed GMNL-653 on human scalp in a clinical trial

**Fig. 4:**
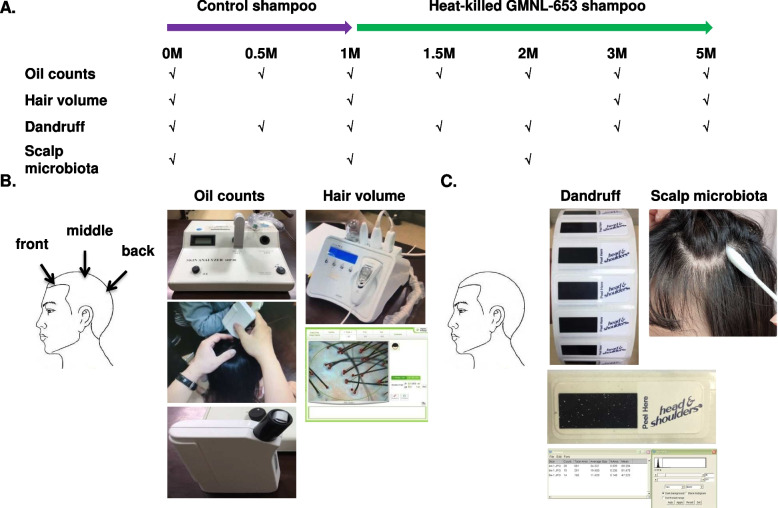
The design of a clinical trial with the application of heat-killed GMNL-653-containing shampoo and examinations of scalp conditions among recruited participants. **A** All participants were requested to use the shampoo without heat-killed GMNL-653 (control shampoo) for the first month. Then, heat-killed GMNL-653-containing shampoo was used for the following 4 month. The collection time points of scalp conditions and scalp microbiota data were indicated by tick. **B** Data of oil count and hair volume were collected from front, middle, and back of scalp using Sebumeter 815 and Aram TSII. **C** Data of dandruff were collected from whole scalp by dandruff tapes and analyzed by Image J. Scalp samples for microbiota analysis were collected from whole scalp by wet cotton swabs containing 1 ml PBS buffer with 0.1% Triton X

Owing to the ability of heat-killed GMNL-653 to suppress *M. furfur* biofilm formation and induce growth factors in skin cells, we examined the beneficial effects of heat-killed GMNL-653-containing shampoo on scalp conditions by conducting a.clinical trial as illustrated in Fig. 4. Twenty-two participants with age ranging 30–45 years and including 8 males and 14 females were recruited. At the first visit, all participants underwent baseline examinations, including oil counts, hair volume, dandruff, and microbiota determination. The baseline of scalp conditions among enrolled participants is summarized in Table 1. At the beginning, all participants were asked to wash their hair by using control shampoo without heat-killed GMNL-653 for 1 month and then change to the tested shampoo with heat-killed GMNL-653 for hair washing for 4 months. Data on oil count, hair volume, dandruff and scalp microbiota were collected from participant scalp according to the timing from the beginning (0M) to the 5th month (5M) as shown in Fig. 4A. The value of oil count was showed by the sum of the three regions (front, middle and back scalp) in each participant, and the value of hair volume was the average from the three regions (Fig. 4B). The samples of dandruff and microbiota were collected from the whole scalp by tape and wet swab, respectively (Fig. 4C), using the methods described in materials and methods.

**Table 1:** Baseline scalp conditions of enrolled participants

**All subjects (*N* = 22)**	
**Age (years)**	**37 ± 6.2**
**Gender (male/female)**	**8/14**
**Scalp conditions**	
**hair volume (normal/low)**	**12/10**
**oil count (normal/high)**	**13/9**
**dandruff (normal/high)**	**12/10**

**Fig. 5:**
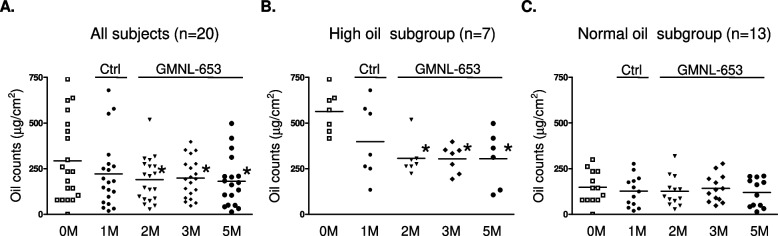
The sebum secretion is decreased in human scalp after using heat-killed probiotics GMNL-653-containing shampoo. Oil counts were collected from all participants scalp at the beginning (0 M), after using control shampoo (Ctrl) for 1 month (1 M), and followed using GMNL-653 containing shampoo (GMNL-653) for 1, 2, and 4 months (2 M, 3 M, and 5 M). Oil counts of the front, middle, and back regions of scalp were detected from each participant and calculated the sum values. **A** Oil counts of all participants (*n* = 20) were shown. **B** Oil counts of 7 participants scalp with high oil condition (> 400 µg/cm^2^) in the beginning were shown. **C** Oil counts from 13 participants with normal oil condition in the beginning were shown.* *p* < 0.05 compared with 0 M group. # *p* < 0.05 compared with the Ctrl group

**Fig. 6:**
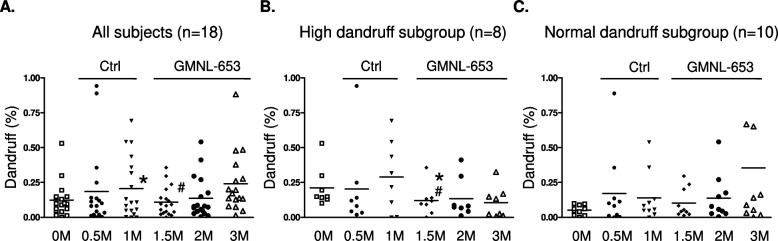
Dandruff formation is decreased in human scalp after using heat-killed GMNL-653-containing shampoo. Dandruff from participants were collected using dandruff tapes in the beginning (0M), after using control shampoo for 0.5 and 1 month (0.5M and 1M), and followed using heat-killed GMNL-653 shampoo for 0.5, 1, and 2 months (1.5M, 2M, 3M). **A** Dandruff level from all participant (*n* = 18) were shown. **B** Dandruff level of 8 participants with high dandruff condition (> 0.1%) at the beginning were shown. **C** Dandruff level of 10 participants with normal condition at the beginning were shown. * *p* < 0.05 compared with 0 M group. # *p* < 0.05 compared with the Ctrl group

**Fig. 7:**
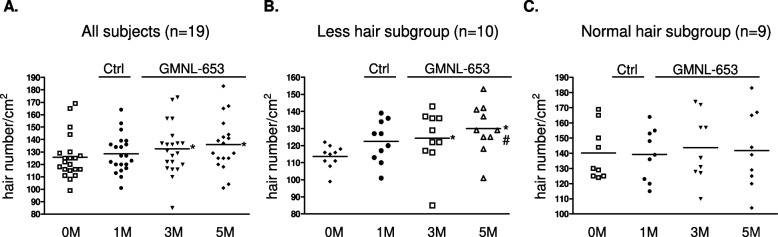
The hair volume is increased in human scalp after using heat-killed GMNL-653-containing shampoo. Hair level from participants were collected at the beginning (0M), after using control shampoo for 1 month (1M), and followed using GMNL-653 containing shampoo for 2 and 4 months (3M and 5M). The front, middle, and back regions of scalp in each participant were detected by Aram TSII. Hair volumes were presented as the sum of value of front, middle, and back scalps from each participant. **A** Hair volumes from all participant (*n* = 19) were shown. **B** Hair volumes of the 10 participants with less hair condition (< 125 hairs/cm^2^) at the beginning were shown. **C** Hair volumes of the 9 participants with normal hair condition (> 125 hairs/cm^2^) at the beginning were shown. * *p* < 0.05 compared with 0 M group. # *p* < 0.05 compared with the Ctrl group

We next performed subgroup analysis according to several scalp conditions, namely, oil situation, dandruff formation, or hair volume. Among the participants, two cases with missing data during the collection time-points were removed. Relative to that at the beginning (0M), the oil count of scalps of 20 subjects after using control shampoo for 1 month (Ctrl; 1M) was not statistical different but was significantly decreased after using heat-killed GMNL-653-containing shampoo for the following 1–4 months (GMNL-653; 2M, 3M, and 5M) (Fig. 5A). Considering that the recruited volunteers initially had various scalp situation, we further classified them into two subgroups of high oil and normal oil depending on their oil situation at the beginning (Start, 0M). We found that the oil count was significantly decreased only in the subgroup of high oil (Fig. 5B), but no statistical difference was observed in the normal oil subgroup (Fig. 5C) after the use of heat-killed GMNL-653-containing shampoo for 1–4 months (GMNL-653; 2M, 3M, and 5M). Among the participants, four cases with missing data during the collection time-points were removed. Relative to that at the beginning (0M), the dandruff formation of 18 subjects statistically increased when they used control shampoo for 1 month (Ctrl; 1M) but significantly decreased when they changed to heat-killed GMNL-653-containing shampoo for 0.5 month (GMNL-653; 1.5M) when compared to the situation at 1 month using control shampoo (Ctrl; 1M) (Fig. 6A). In the subgroup comparisons, dandruff formation was significantly reduced only in the subgroup with high dandruff (Fig. 6B) but not in the subgroup with normal dandruff (Fig. 6C) after the use of heat-killed GMNL-653 shampoo for 0.5 month (GMNL-653; 1.5M) compared with that at the beginning (0M) and using control shampoo for 1 month (Ctrl; 1M). For hair volume analysis, only 19 subjects were analyzed after removing 3 cases with missing data during the collection time-points. The hair volume statistically increased after using heat-killed GMNL-653 shampoo for 2 and 4 months (GMNL-653; 3M, 5M) compared with that at the beginning (0M) among all subjects (Fig. 7A). In the subgroup analysis, the increase in hair level with time caused by using heat-killed GMNL-653 shampoo was observed only in the subgroup with low hair abundance (Fig. 7B) but not in the subgroup of normal hair abundance (Fig. 7C).

### Changes of microbiota in human scalp after using heat-killed GMNL-653-containing shampoo

**Fig. 8:**
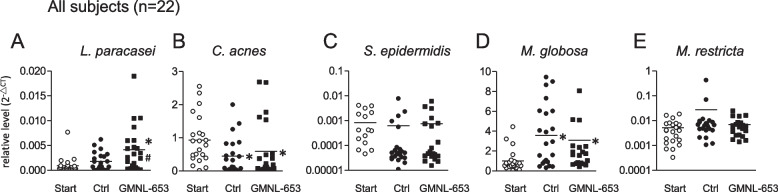
The abundance of fungal and bacterial microbiota on human scalp after using heat-killed GMNL-653-containing shampoo. Microbiota samples of each participant were collected from the whole scalp by wet cotton swabs with 0.1% Triton X /PBS buffer at the beginning (Start), after using control shampoo for 1 month (Ctrl), and followed using GMNL-653 shampoo for 1 months (GMNL-653). The liquid samples in cotton swabs were centrifuged to collect the pellets followed by extracting DNA for microbiota analysis. The accumulation of *L. paracasei* (**A**) and the abundance of *C. acnes* (**B**), *S.epidermidis* (**C**), *M. globosa* (**D**), and *M. restricta* (**E**) in Start, Ctrl, and GMNL-653 groups were quantified by qPCR method. The relative quantified data of *L. paracasei*, *C. acnes*, and *S. epidermidis* were normalized with total bacteria value (**A**-**C**) and *M. globosa* and *M. restricta* were normalized with total *Malassezia* value (**D**-**E**). * *p* < 0.05, and ** *p* < 0.01 compared with Start group. # *p* < 0.05 compared with the Ctrl group

We focused on the two bacteria, *C. acnes* and *S. epidermidis*, and two fungi, *M. restricta* and *M. globosa*, to analyze the scalp microbial level of participants by quantitative PCR before or after using control and heat-killed GMNL-653 shampoo. First, we confirmed that the level of *L. paracasei* from the scalp samples of all participants significantly increased after using heat-killed GMNL-653 shampoo relative to that at the beginning (Start) and after using the control shampoo (Ctrl) using (Fig. 8A). We further observed that the abundance of *C. acnes* statistically decreased (Fig. 8B) and that of *M. globosa* statistically increased (Fig. 8D) after using heat-killed GMNL-653 shampoo relative to that at the beginning (Start). However, the level of *S. epidermidis* (Fig. 8C) and *M.* *restricta* (Fig. 8E) among all scalp samples did not show statistical differences. The decreasing level of *C. acnes* (Fig. 8B) and the increasing level of *M. globosa* (Fig. 8D) were observed in the group using control shampoo (Ctrl) relative to that at the beginning (Start), indicating that the control shampoo had the same effect as heat-killed GMNL-653 shampoo on the abundance of *C. acnes* or *M. globose*. We further analyzed the microbial abundance relative to that at the beginning scalp condition after classifying the participants into weak and normal subgroups as described above. In the three kinds of scalp situations (dandruff, oil secretion, and hair volume), the increased accumulation of *L.* *paracasei* was observed in the three normal subgroups (Supplementary Figs. 1A, C, and E) and in the subgroup with less hair (Supplementary Fig. 1F) after the use of heat-killed GMNL-653 shampoo. The abundance of *M. restricta* was not different among all subjects (Fig. 8E) but statistically decreased in the subgroup of high dandruff after the use of heat-killed GMNL-653 shampoo (Supplementary Fig. 1B). In addition, a decrease in *C. acnes* in the high dandruff subgroup and normal subgroups of oil and hair (Supplementary Figs. 1B, C, and E) and an increase in *M. globosa* in the normal subgroups of dandruff, oil, and hair (Supplementary Figs. 1A, C, and E) were observed regardless of using control or heat-killed GMNL-653 shampoo. However, the abundance of *C. acnes* in the subgroups of normal dandruff, high oil, and less hair (Supplementary Figs. 1A, 1D, and 1F) and the abundance of *M. globosa* in the three weak subgroups of high dandruff, high oil, and less hair (Supplementary Figs. 1B, D, and F) showed no statistical difference after the use of heat-killed GMNL-653 shampoo. These results suggest that the beneficial effects of heat-killed GMNL-653 shampoo on human scalp health may be related to the changes of scalp microbiota.

### Correlation between microbial species in human scalp microbiota after using heat-killed GMNL-653 shampoo

We then investigated the association between the increase in *L. paracasei* on the scalp and four commensal microbes, namely, *M. restricta*, *M. globosa*, *C. acnes*, and *S. epidermidis*. Sixty-six data entries collected from three different time points of 0, 1, and 2 months during the clinical trial were used for analysis. The results showed that *L. paracasei* level was negatively correlated with that of *C. acnes* and positively correlated with that of *M. globosa* but was not statistically correlated with that of *S. epidermidis* and *M.* *restricta* (Table 2). We further observed the correlations in the normal and weak subgroups of the three scalp conditions, namely, hair growth, oil secretion, and dandruff. Similarly, *L. paracasei* abundance was negatively correlated with that of *C. acnes* and positively correlated with that of *M. globosa* in the normal subgroups of hair, oil, and dandruff. *L.* *paracasei* abundance was statistically positively correlated with that of *S. epidermidis* in the high dandruff subgroup. However, no statistically correlation was observed between the abundances of *L. paracasei* and *M. restricta* even in all subgroups (Table 2). Next, we determined the correlations among the two fungal and two bacterial species in the clinical trial of using heat-killed GMNL-653 shampoo. Table 3 lists 66 collected data used to observe the correlation between bacteria and fungus. *S. epidermidis* abundance was negatively correlated with that of *M. globosa* and positively correlated with that of *M. restricta*. *C. acnes* abundance was negatively correlated with that of *M. globosa* but not statistically correlated with that of *M. restricta* according to the 66 data observation. In the subgroup observation, *S. epidermidis* abundance was negatively correlated with that of *M. globosa* in the weak subgroup of low hair. A positive correlation between *S. epidermidis* and *M. restricta* abundance and a negative correlation between *C. acnes* and *M. globosa* abundance were observed in the weak subgroup of low hair and in the normal subgroups of oil and dandruff. For the correlation between two fungi, *M. globosa* abundance was positively correlated with that of *M. restricta* in the high dandruff subgroups. However, no statistical correlation was found between bacterial *S. epidermidis* and *C. acnes* abundance in total data and in the subgroups of three scalp conditions. These data suggest that the use of heat-killed GMNL-653 shampoo could change the scalp microbiome to achieve its beneficial effects on scalp health.

**Table 2:** Correlations of *L. paracasei* level with* C. acnes*,* M. globosa*,* S. epidermidis*, or* M. restricta* in the shampoo clinical trial

Correlation between	All (*n* = 66)	Hair		Oil		Dandruff	
		Normal (*n* = 36)	Low (*n* = 30)	Normal (*n* = 45)	High (*n* = 21)	Normal (*n* = 42)	High (*n* = 24)
*L. paracasei* and *C. acnes*							
Pearson’s r	-0.29	-0.405	-0.019	-0.311	-0.226	-0.312	-0.257
*P* value	0.018*	0.014*	0.919	0.037*	0.332	0.044*	0.225
*L. paracasei* and *M. globosa*							
Pearson’s r	0.417	0.453	0.273	0.489	0.045	0.524	0.047
*P* value	0.001**	0.006**	0.152	0.001**	0.85	< 0.001***	0.832
*L. paracasei* and* S. epidermidis*							
Pearson’s r	-0.095	0.21	-0.36	-0.207	0.384	-0.218	0.526
*P* value	0.446	0.218	0.051	0.172	0.086	0.166	0.008**
*L. paracasei* and *M. restricta*							
Pearson’s r	-0.078	0.055	-0.164	-0.09	-0.178	-0.1	0.32
*P* value	0.537	0.748	0.396	0.557	0.453	0.53	0.136

^*^ means *P* < 0.05 and ** means *P* < 0.01, *** means *P* < 0.001

**Table 3:** Correlations among the fungal and bacterial species in the shampoo clinical trial

Correlation between	All (*n* = 66)	Hair		Oil		Dandruff	
		Normal (*n* = 36)	Low (*n* = 30)	Normal (*n* = 45)	High (*n* = 21)	Normal (*n* = 42)	High (*n* = 24)
*S. epidermidis* and* M. globosa*							
Pearson's r	-0.258	-0.104	-0.377	-0.263	-0.22	-0.298	-0.166
*P* value	0.038*	0.547	0.044*	0.081	0.351	0.055	0.449
*S. epidermidis* and *M. restricta*							
Pearson's r	0.586	-0.032	0.654	0.683	0.184	0.612	0.063
*P* value	< 0.001**	0.851	< 0.001***	< 0.001***	0.436	< 0.001***	0.766
*C. acnes* and* M. globosa*							
Pearson's r	-0.337	-0.306	-0.431	-0.322	-0.377	-0.325	-0.365
*P* value	0.006**	0.069	0.019*	0.031*	0.101	0.035*	0.086
*C. acnes* and* M. restricta*							
Pearson's r	-0.1	-0.011	-0.167	-0.135	0.586	-0.125	-0.13
*P* value	0.426	0.948	0.386	0.376	0.007**	0.431	0.555
*M. globosa* and *M. restricta*							
Pearson's r	-0.107	-0.058	-0.147	-0.129	0.07	-0.137	0.451
*P* value	0.395	0.739	0.446	0.398	0.768	0.388	0.031*
*C. acnes* and *S. epidermidis*							
Pearson's r	0.135	-0.008	0.316	0.088	0.204	0.147	0.119
*P* value	0.278	0.965	0.089	0.563	0.376	0.354	0.579

^*^ means *P* < 0.05, ** means *P* < 0.01, *** means *P* < 0.001

### Potential role of bacterial shifts in human scalp health

**Fig. 9:**
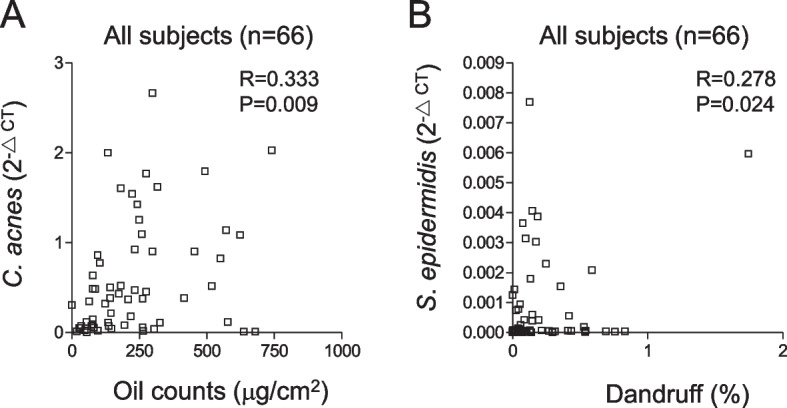
The correlations of *C. acnes* or *S. epidermidis* to human scalp conditions after using heat-killed GMNL-653-containing shampoo. The levels of *C. acnes* and *S. epidermidis* of all participants in three time points after using heat-killed GMNL-653 shampoo (0, 1, 2 months, *n* = 66) were used for analyzing the Pearson correlation coefficient (R) to oil counts. R and the *p* value were calculated by SPSS statistics

We investigated whether the microbiota shift modulated by heat-killed GMNL-653 influences the scalp condition and health. By analyzing the correlations of microbial abundance with the three scalp conditions, namely, hair growth, sebum secretion and dandruff, we found that the abundance of bacterial *C. acnes* and *S. epidermidis* was positively correlated with sebum secretion and dandruff, respectively, after the use of GMNL-653 shampoo for 2 months (Fig. 9). However, the two bacteria were not correlated with hair growth of the 3rd and 5th months (Supplementary Table S2). The abundance of fungi *M. restrica* and *M. globosa* did not show statistical correlations with sebum secretion, hair growth, and dandruff (Supplementary Table S2). These results indicate that the beneficial effects of heat-killed GMNL-653-containing shampoo on sebum secretion and dandruff are correlated with the changes of *C. acnes* and *S. epidermidis*.

## Discussion

Reygagne et al*.* [26] reported that orally consumed active probiotics *L. paracasei* NCC2461 ST11 could restore human severe dandruff by modulating the scalp microbiota. Topical cream containing heat-killed *L. platarum* GMNL-6 also improves human skin health [23]. We previously demonstrated that the treatment of smeared gel containing heat-killed *L. platarum* GMNL-6 and *L.* *paracasei* GMNL-653 on wounded mouse tails exhibited excellent healing ability and the capacity to prevent excessive fibrosis through the suppression of TGF-β/pSmad signaling in the skin wound repair [22]. In the current study, we found that heat-killed *L. paracasei* GMNL-653 could ameliorate damaged human scalp health for the first time. Improvements in human scalp conditions including decreased oil secretion and dandruff formation and increased hair volume were observed after the use of heat-killed GMNL-653-containing shampoo (Figs. 5, 6 and 7). In particular, the effect of heat-killed GMNL-653 shampoo on improving scalp health was observed for the participants with weak scalp condition at the beginning, such as the weak subgroups of high oil (Fig. 5B), high dandruff (Fig. 6B), and less hair (Fig. 7B), but not apparent for the participants with the healthy scalp condition (Figs. 5C, 6C, and 7C). The results suggest that the beneficial effect of the heat-killed GMNL-653 shampoo is more significant for the people with weak scalp conditions than for those with healthy conditions.

We also showed that heat-killed *L. paracasei* GMNL-653 could co-aggregate with *M. furfur*, and GMNL-653-derived LTA could inhibit the biofilm formation of *M. furfur* in our in vitro study (Fig. 1). Adherence, hydrophobicity, and biofilm formation are important virulence factors to change skin *M. furfur* from commensal status to pathogen status [27], and the pathological role of *M. furfur* is strongly correlated with dandruff formation in human scalp [28]. However, our in vivo studies for *M. furfur* could not demonstrate the correlation (data not shown). This phenomenon can be attributed to the fact that our clinical project recruited participants with healthy scalp condition rather than those with scalp diseases. Other studies showed that the distribution of *Malassezia* species in scalps of different populations could vary depending on factors such as differences of scalp disease, age, gender, body site, and resident region [29–31]. For example, one study found that the isolation frequency of *M. furfur* was 13% in healthy subjects, 25% in patients with SD, 10% in patients with atopic dermatitis, and 52.5% in patients with pityriasis versicolor [29]. The specific population of recruited participants must be considered to study the correlation of *M. furfur* with scalp condition in the future. Our in vivo observation also showed that the scalp microbial abundance of fungus *M. restricta* and *M. globosa* or bacteria *C. acnes* and *S.* *epidermidis* was observed after the participants with normal scalp condition at the beginning used heat-killed GMNL-653 shampoo (Fig. 8). *M. restricta* and *M. globosa* are the predominated species in human scalp *Malassezia* colonization. In particular, *M. restricta* has a critical role in the pathogenesis of dandruff and SD among the different species of *Malassezia* [32–35]. Recent studies from French, Indian, Chinese, and Korean populations demonstrated that *M. restricta* is more abundant in scalps with dandruff disease than in healthy scalps [8, 36–38]. *M. restricta* lipases of MrLip1 or MrLip2 could hydrolyze mono- and diacylglycerol, and MrLip3 could hydrolyze mono-, di, and tri-acylglycerol [39]. MrLip5 lipase plays an essential role in *M. restricta*-host interfere during disease progression, and the MrLip5 lipase encoded by the genome of *M. restricta* shows stable activity and high expression under alkaline condition [40]. In our study, the abundance of *M. restricta* was not statistically different among the scalp samples collected before and after the use of heat-killed GMNL-653 shampoo in all subjects (Fig. 8E). However, subgroup analysis revealed that *M. restricta* abundance was statistical decreased in the weak subgroup of high dandruff after the use of heat-killed GMNL-653 shampoo (Supplementary Fig. 1B), suggesting that GMNL-653 could decrease pathogenic fungus in dandruff scalp. Although the role of *M. globosa* in the scalp is inconsistent [41–43], it has beneficial effect on dandruff scalp [8, 15, 42]. In this study, we found that *M. globosa* abundance was up-regulated in all subjects (Fig. 8D) and in the normal subgroups of scalp conditions (Supplementary Figs. S1A, C and E) after the use of heat-killed GMNL-653 shampoo. This finding indicates that *M. globosa* plays a beneficial role in scalp health in our shampoo model. How heat-killed GMNL-653 can positively and negatively regulate different *Malassezia* species in the scalp must be investigated in the future.

*C. acnes* and *S. epidermidis* are the most abundant commensal bacteria in human scalp, and their diversities are strongly correlated with scalp disorder and diseases. Grimshaw et al*.* [9] found that the abundance of *C. acnes* is associated with healthy scalp and that of *S. epidermidis* is associated with dandruff scalp. They also found that the ratio of *C. acnes* to *S. epidermidis* abundance is higher in healthy scalp than in dandruff scalp. However, our results showed that *C. acnes* abundance was down-regulated after the use of heat-killed GMNL-653 shampoo (Fig. 8B). We suggest that the role of *C. acnes* in our model of heat-killed GMNL-653 shampoo treatment might be contrary and different from that in the dandruff scalp. Other studies also provided evidence that the role of *C. acnes* is correlated with hair growth disorder [44–47]. For example, elevated *C. acnes* in the middle and lower compartments of hair follicles are associated with elevated immune response in patients with alopecia areata [45, 46]. A significant increase in *C. acnes* and a decrease in *S. epidermidis* were also observed in patients with alopecia areata [44] and androgenetic alopecia [47] compared with those in control. Further investigation of the potential role of *C. acnes* in the regulation of scalp hair growth under heat-killed GMNL-653 treatment using an in vitro culture of human hair follicles or in mouse models is necessary.

Microbiome dysbiosis is significantly correlated with skin disease [48, 49], indicating that a balanced microbiome is important to maintain the local cutaneous health. Although Xu et al*.* [7] indicated that bacteria (*Cutibacterium* and *Staphylococcus*) and fungi (*Malassezia*) did not show a close association with each other in dandruff scalp, our study shows new evidence that bacteria could be associated with fungus on scalp. In our clinical observation, we found that the abundance of *S. epidermidis* was negatively correlated with that of *M. globosa* and positively correlated with that of *M. restricta*. The same trends were observed for *C. acnes* with *M. globosa* and *M. restricta* (Table 3). Another study reported the interaction between bacteria and fungus. The co-colonization of two dominant scalp microbes, *M. restricta* and *C. acnes,* in 3D reconstructed human epidermis model could establish a community and a homeostasis interaction with host, improving the barrier damage and structural degradation that happened in the single colonization of *M. restricta* [50]. The correlation between bacteria and fungusmay serve as the indicator for microbiome balancing and equilibrium in the scalp after the use of heat-killed GMNL-653 shampoo. Our study also found a negative correlation between *L. paracasei* and *C.* *acnes* abundance and a positive correlation between *L. paracasei* and *M. globosa* abundance (Table 2). This finding suggests that *L. paracasei* GMNL-653 may regulate skin microbiota network through direct or indirect manner. However, we only recruited a small number of participants with healthy scalp to observe the correlation among the four predominant microbes (*M. restrica*, *M. globosa*, *C. acnes*, and *S. epidermidis*). Other bacteria or fungus may also be changed after application of heat-killed GMNL-653-containing shampoo. Next-generation sequencing of bacterial 16S rRNA or fungal Internal Transcribed Spacer rRNA amplicons should be applied in the future to identify the whole change of microbiome involved in the beneficial effect of heat-killed GMNL-653 on scalp health.

Our finding showed that LTA purified from GMNL-653 inhibited the biofilm formation of *M. furfur* (Fig. 1D), which was consistent with our previous studies on *Staphylococcus aureus* [22, 23]. The LTA structure varies among different bacterial species [51]. LTAs from various *L. plantarum* strains also have different effects on anti-biofilm for dental pathogenic bacteria [52]. Our previous study observed that GMNL-653 uniquely expresses six genes involved in cell wall/membrane/envelop biogenesis compared with the other two *L. paracasei* strains (GMNL-855 and BCRC 16100). These genes are involved in glycan biosynthesis and metabolism, lipopolysaccharide cholinephosphotransferase, and fructoselysine 6-phosphate deglycase [24]. Whether these six uniquely expressed genes are involved in the strain-specific structure of GMNL-653 LTA that contributes to the anti-biofilm activity against scalp pathogens is worthy of further investigation.

On the basis of our in vitro observations presented in Figs. 2 and 3, the direct treatment of heat-killed *L. paracasei* GMNL-653 on human skin cells up-regulated the gene expressions of hair follicle growth factors including IGF-1R, VEGF, KGF, and IGF, which are involved in the regeneration system of hair follicle cycling [53]. The benefits of heat-killed GMNL-653 were also observed in the in vivo clinical trial, indicating that the accumulation of *L. paracasei* was positively correlated with the hair volume after the participants used heat-killed GMNL-653 shampoo for 2 and 4 months (Supplementary Fig. S2). These findings suggest that heat-killed *L. paracasei* GMNL-653 has the potential to improve scalp condition and enhance hair growth. Several studies reported that an increase in *C. acnes* abundance is strongly associated with hair growth disorders, such as alopecia areata and androgenetic alopecia [44, 46, 47]. Our results also showed that the use of heat-killed GMNL-653 shampoo could decrease the abundance of *C. acnes* in scalp (Fig. 8D). The reduction in *C. acnes* abundance caused by heat-killed GMNL-653 shampoo would be the key point to regulate the hair growth and improve the hair loss in scalp.

## Conclusions

We demonstrate that the application of heat-killed *L. paracasei* GMNL-653-containing shampoo in the scalp could improve the scalp condition and modulate scalp microbiota. The changes of microbiota diversity induced by heat-killed GMNL-653 could be a potential mechanism in regulating the scalp conditions, such as the control of sebum secretion, dandruff generation, and hair growth.

## Materials and methods

### Bacterial culture and heat-inactive preparations

*L. paracasei* GMNL-653 (BCRC-910721, CCTCC M2016226) were provided by GenMont Biotech (Tainan, Taiwan). GMNL-653 was cultured in 1 ml of MRS broth (Neogen Corporation, Lansing, MI, USA) at 37 °C for 20 h and subcultured at the ratio of 1:100 in MRS medium at 37 °C for another 20 h. The cells were then centrifuged at 13,000 rpm for 1 min, and cell pellets were washed with PBS and finally suspended in PBS. The cell number of bacteria was adjusted to the concentration of 2 × 10^9^–1 × 10^10^ cells/ml for further experiments. *Malassezia furfur* (BCRC-22243, obtained from Bioresource Collection and Research Center, Hsinchu City, Taiwan) was cultured on modified Leeming–Notman agar plate at 30 °C incubator for 2 days and then subcultured. After secondary activation, the bacteria were collected from agar plates and suspended in PBS with the concentration adjusted to 2 × 10^9^ cells/ml. For the heat-killed preparation of GMNL-653, bacterial cells were suspended as 1 × 10^10^ cells/ml and subjected to an autoclave at 121 °C for 15 min.

### Purification of lipoteichoic acid (LTA)

After PBS washing and centrifugation, the cell number of GMNL-653 was adjusted to the concentration of 1 × 10^10^ cells/ml, followed by heat-inactive preparation using autoclave at 121 °C for 15 min. GMNL-653 cells were then disrupted by the machine of ultrasonic disruptor (ultrasonic instrument model W-220F, Bioventus LLC., Farmingdale, NY, USA) and then centrifuged. The bacteria were reconstituted in the mixture of chloroform–methanol-water (1:1:0.9), and the aqueous phase containing LTA was collected. The LTA-containing solution was injected into Octyl-Sepharose CL-4B column (Sigma-Aldrich, Saint Louis, MO, USA) and Q-Sepharose column (Sigma-Aldrich) for further purification, followed by freeze-drying. The LTA powder was then dissolved in water at a concentration of 5 mg/ml, filtered using a 0.22 μm syringe filter (Sigma-Aldrich), and stored at − 20 °C.

### Co-aggregation test and scanning electron microscopy (SEM)

Heat-killed preparation of GMNL-653 (2 × 10^9^ cells/ml) was mixed with *M. furfur* (2 × 10^9^ colony-forming unit (CFU)/ml) in the ratio of 1:1 in test tube. After mixing for 30 min, the fibrous-like aggregates became visible. Aggregation in each of the mixtures was observed at 0, 20, and 40 min. Co-aggregation ability was calculated as described by Fukao et al. [54] with slight modification. After GMNL-653 was incubated with *M. furfur* at different durations, the OD of 590 nm from the supernatant of each tube was detected. The OD_590_ of supernatant from *M. furfur* alone tube, GMNL-653 alone tube, and *M. furfur*-GMNL mixture tube was represented as OD_*M. furfur*_, OD_GMNL-653_, and OD_Mixture_, respectively. Co-aggregation ability was measured using the following equation: Co-aggregation ability (OD_590_) = OD_*M. furfur*_ + OD_GMNL-653_ − OD_Mixture_. The co-aggregated pellets of heat-killed GMNL-653 and lived *M. furfur* were rinsed with PBS buffer to remove unattached cells. After fixation, dehydration, and coating with gold, the aggregated samples were detected by SEM in the Instrument Center in National Cheng Kung University (Tainan City, Taiwan).

### Cell culture

Human foreskin fibroblast Hs68 cells and human epidermal keratinocyte HaCaT cells were obtained from American Type Culture Collection (ATCC, Manassas, VA, USA) and cultured in Dulbecco’s modified Eagle medium supplemented with 10% fetal bovine serum, 100 U/ml penicillin, and 100 μg/ml streptomycin under steady-state conditions at 37 °C and 5% CO_2_ in the humidified incubator.

#### Biofilm formation assay

*M. furfur* was seeded into the wells of a 96-well-plate (2 × 10^8^ CFU/well) with or without GMNL-653-derived LTA (25 or 50 μg/ml) for 24 h. After the supernatants were removed, the wells were gently washed twice with PBS to remove unattached bacterial cells. The biofilm was fixed with 95% ethanol for 10 min and stained with 0.1% crystal violet for 15 min. After being washed with PBS three times, the crystal violets were dissolved with 10% acetic acid quantified by the absorbance at a wavelength of 590 nm.

### Analysis of the expression of growth factors by RNA isolation and quantitative reverse transcription-polymerase chain reaction (qRT-PCR)

Hs68 cells (1.5 × 10^5^ cells/well) or HaCaT cells (3 × 10^5^ cells/well) were cultured in six-well plates for 24 h. After being washed twice with PBS, the cells in the wells were cultured with serum-free medium for 24 h and then treated with different concentrations of heat-killed GMNL-653 (ranged from 0.3125 × 10^8^ to 5 × 10^8^ cells/well) for 24 h. Total cellular RNA was extracted by TRIzol reagent (Thermo Fisher Scientific, Waltham, MA, USA). In brief, 5 μg of RNA was used for cDNA synthesis with RevertAid RT Reverse Transcription Kit (Thermo Fisher Scientific). qRT-PCR was employed to analyze the mRNA expression of hair-associated growth factors including IGF-1R, IGF-1, VEGF, and KGF with the following steps: the reaction mixtures were prepared by adding 5 μl of 2 × Rotor-Gene SYBR Green PCR Master Mix (204,076; QIAGEN, Hilden, Germany), 2 μl of cDNA and 3 μl of forward and reverse primers to a final volume of 10 μl. The reaction mixtures were analyzed on the Roter-Gene Q 2plex machine (QIAGEN). Quantitative values were calculated from the threshold cycle (Ct) number with the equation as 2^−△△Ct^, where △△Ct is △Ct_treatment group_ – △Ct_control group_. □-actin (ACTB) was used as the housekeeping gene. Control group indicated that the cells were treated without heat-killed GMNL-653. The sequences of primers (5’ to 3’) are listed in Table S1.

### Study design of clinical trial

A 5-month clinical study was approved by the Institutional Review Board in Antai-Tian-Sheng Memorial Hospital (TSMH IRB No./ Protocol No. 20–040-A, approval date: 06/07/2020), Antai Medical Care Cooperation, Donggang Township, Pingtung County, Taiwan) and performed in accordance with the relevant guidelines and regulations. Informed consent was obtained from all subjects and/or their legal guardians. The study was registered at ClinicalTrials.gov with approval number NCT04566549 (first posted date: 28/09/2020), and the data were collected at Chia Nan University of Pharmacy and Science (Tainan, Taiwan). Twenty-two healthy adults (age: 37 ± 6.2 years old; 8 males and 14 females) were recruited (Table 1). The participants underwent examinations including oil count, hair volume, dandruff, and scalp microbiota, the primary outcomes of this trial, according to the time points of using shampoo shown in Fig. 4. In the first month, all participants used the baseline control shampoo without heat-killed probiotics GMNL-653 to wash their hair. The ingredients of control shampoo contained aqua, sodium lauryl ether sulphate, cocamidopropyl betaine, cocoamide DEA, sodium, polyquaternium-10, disodium EDTA, acrylates copolymer, citric acid, phenoxyethanol, and fragrance. Afterward, the participants washed hair with heat-killed probiotics GMNL-653-containing shampoo for 4 months. This shampoo contained 0.5% heat-killed *L. paracasei* and the same ingredients found in the control shampoo. The frequency of hair washing would be once in each day or twice each day depending on their personal habits. For the checking of subjects’ compliance, the average use dosage of control shampoo was 152 ± 69.8 g/month and GMNL-653-containing shampoo was 161.5 ± 56.2 g/months. The application status of two kinds of shampoos in 22 subjects were similar (*P-*value = 0.812). The value of oil count was the sum of three regions of scalp (front, middle, and back) analyzed by Sebumeter 815 (Courage + Khazaka electronic GmbH, Köln, Germany) in each participant. The hair volume was the average of three scalp regions (front, middle, and back) analyzed and measured by Aram TSII (Visia Complexion Analysis, Canfield Scientific, Inc., Parsippany, NJ, USA). For the test of dandruff, the flakes were collected from the whole scalp by dandruff D-Squame tape (Clinical and Derm LLC., Dallas, TX, USA). The dandruff was evaluated by measuring the percentage of flakes in the total area of dandruff tape using Image J software (version 1.53n 7, National Institutes of Health, Bethesda, Maryland, USA). The scalp conditions of hair volume, oil secretion, and dandruff were classified into normal and weak subgroups according to the scalp situation at the beginning without shampoo treatment. The subgroups of hair volume (normal: > 125 hairs/cm^2^; weak: > 125 hairs/cm^2^), oil secretion (normal: < 400 μg/cm^2^; weak: > 400 μg/cm^2^), and dandruff (normal: < 0.1%; weak: > 0.1%) were defined. Each value of oil level was the sum of three measured sites from front, middle, and back scalp.

### DNA extraction and analysis of scalp microbiota by quantitative PCR (qPCR)

For the analysis of scalp microbiota, samples of whole scalp were collected by wet cotton swab containing 1 ml of PBS buffer with 0.1% Triton X-100. The separate areas of scalp including front left, front right, back left, back right, edge of the hair line, and behind the ears were swabbed following the Z line back and forth at least three times. The swab head was placed in a 1.5 ml Eppendorf tube to vortex for 30 s and incubated in room temperature for 15 min. After incubation, the swab head was centrifuged at 13,000 rpm for 10 min and then transferred to sterile H_2_O. The samples were dissolved and released out by swirling and snapping the swab. After the swab was removed, the liquid sample was centrifuged at 13,000 rpm for 5 min and the supernatant was then removed. DNA from the pellets was extracted by Quick-DNA fungal/bacterial kit (Zymo Research Corporation, Irvine, CA, USA). The reaction mixtures were prepared by 5 μl of 2 × Rotor-Gene SYBR Green PCR Master Mix (QIAGEN), 2 μl DNA from scalp extract and 3 μl of forward and reverse primers. The primer sequences of target microbes and internal control (total bacteria for *L. paracasei*, *C. acnes*, or *S. epidermidis* or total *Malassezia* for *M. globose* or *M. restricta*) are shown in part B of Table S2. The reaction mixtures were analyzed on the Roter-Gene Q 2plex machine (QIAGEN). The relative quantification of changes in specific bacterial species were calculated by the equation 2^−△Ct^, where △Ct is Ct_target bacteria_ – Ct_total bacteria_. The relative quantification of changes in specific *Malassezia* species were calculated by the equation 2^−△Ct^, where △Ct is Ct_target *malassezia*_ – Ct _*malassezia*_.

### Statistical analysis

All data were presented as mean ± SEM. Comparisons between two groups were determined using unpaired *t*-test, and comparisons among three groups or above were performed by one-Way ANOVA and Tukey’s post hoc tests. Pearson correlations between two microbiota variables were computed by PASW Statistical 18 Software (SPSS Inc., Chicago, IL, USA). A *p* value less than 0.05 was considered as statistically significant.

## Supplementary Information

**Additional file 1: Table S1.** The primer sequences of hair follicle growth factor-related genes (A) and hair follicle microbiota-related genes (B). **Table S2.** Correlations of the fungus or the bacteria with scalp conditions in the shampoo clinical trial. **Figure S1.** Heat-killed *L. paracasei* GMNL-653 modulates microbiota diversity in the subgroups of normal dandruff (A), high dandruff (B), normal oil (C), high oil (D), normal hair (E), and less hair (F). Start group (Start) described as the scalp in the beginning without any shampoo treatment. Control group (Ctrl) represented as the use of control shampoo without adding heat-killed GMNL-653 for 1 month. GMNL-653 group (GMNL-653) meant the use of heat-killed GMNL-653 shampoo for 1 month after using Ctrl shampoo. * *p* < 0.05 compared with Start group. # *p* < 0.05 compared with the Ctrl group. **Figure S2.** Hair volume is positive-correlated with accumulation of *L. paracasei* in human scalp after using heat-killed GMNL-653 shampoo for 2 months (A) and 4 months (B). Pearson correlation coefficient (R) and *p* value were calculated by SPSS statistics. 

## Data Availability

All the primer sequences were provided in Supporting information (Table S1). There is no data to deposit in a database and no sequencing has been performed during the current study. To request the data from this study, please contact with CWW (changww@csmu.edu.tw).

## References

[1] Chen YE, Fischbach MA, Belkaid Y (2018). Skin microbiota-host interactions. Nature.

[2] Polak-Witka K, Rudnicka L, Blume-Peytavi U, Vogt A (2020). The role of the microbiome in scalp hair follicle biology and disease. Exp Dermatol.

[3] DeAngelis YM, Gemmer CM, Kaczvinsky JR, Kenneally DC, Schwartz JR, Dawson TL (2005). Three etiologic facets of dandruff and seborrheic dermatitis: Malassezia fungi, sebaceous lipids, and individual sensitivity. J Investig Dermatol Symp Proc.

[4] Trüeb RM, Henry JP, Davis MG, Schwartz JR (2018). Scalp condition impacts hair growth and retention via oxidative stress. Int J Trichology.

[5] Suzuki K, Cho O, Nagahama T, Sugita T (2022). Short sequence repeats of the intergenic spacer regions of ribosomal RNA genes in Malassezia globosa and Malassezia restricta colonizing the scalps of male individuals with and without androgenetic alopecia. Microbiol Immunol.

[6] Constantinou A, Kanti V, Polak-Witka K, Blume-Peytavi U, Spyrou GM, Vogt A (2021). The potential relevance of the microbiome to hair physiology and regeneration: the emerging role of metagenomics. Biomedicines.

[7] Xu Z, Wang Z, Yuan C (2016). Dandruff is associated with the conjoined interactions between host and microorganisms. Sci Rep.

[8] Saxena R, Mittal P, Clavaud C (2018). Comparison of healthy and dandruff scalp microbiome reveals the role of commensals in scalp health. Front Cell Infect Microbiol.

[9] Grimshaw SG, Smith AM, Arnold DS, Xu E, Hoptroff M, Murphy B (2019). The diversity and abundance of fungi and bacteria on the healthy and dandruff affected human scalp. PLoS One.

[10] Polak-Witka K, Constantinou A, Schwarzer R (2021). Identification of anti-microbial peptides and traces of microbial DNA in infrainfundibular compartments of human scalp terminal hair follicles. Eur J Dermatol.

[11] Kayıran MA, Sahin E, Koçoğlu E, Sezerman OU, Gürel, MS, Karadağ AS. Is cutaneous microbiota a player in disease pathogenesis? Comparison of cutaneous microbiota in psoriasis and seborrheic dermatitis with scalp involvement. Indian J Dermatol Venereol Leprol. 2022;88(6):738–48. 10.25259/IJDVL_323_21 35389020

[12] Soares RC, Zani MB, Arruda AC, Arruda LH, Paulino LC (2015). Malassezia intra-specific diversity and potentially new species in the skin microbiota from Brazilian healthy subjects and seborrheic dermatitis patients. PLoS One.

[13] Jain S, Arora P, Nainwal LM (2021). Essential oils as potential source of anti-dandruff agents: a review. Comb Chem High Throughput Screen.

[14] Lourith N, Kanlayavattanakul M, Chaikul P (2021). Para rubber seed oil: the safe and efficient bio-material for hair loss treatment. J Cosmet Dermatol.

[15] Saxena R, Mittal P, Clavaud C (2021). Longitudinal study of the scalp microbiome suggests coconut oil to enrich healthy scalp commensals. Sci Rep.

[16] Algburi A, Alazzawi SA, Al-Ezzy AIA, Weeks R, Chistyakov V, Chikindas ML (2020). Potential probiotics *Bacillus*
*subtilis* KATMIRA1933 and *Bacillus*
*amyloliquefaciens* B-1895 co-aggregate with clinical isolates of proteus mirabilis and prevent biofilm formation. Probiotics Antimicrob Proteins.

[17] Prescott SL, Larcombe DL, Logan AC (2017). The skin microbiome: impact of modern environments on skin ecology, barrier integrity, and systemic immune programming. World Allergy Organ J.

[18] Park DW, Lee HS, Shim MS, Yum KJ, Seo JT (2020). Do kimchi and *cheonggukjang* probiotics as a functional food improve androgenetic alopecia? A clinical pilot study. World J Mens Health.

[19] Yoon YC, Ahn BH, Min JW, Lee KR, Park SH, Kang HC (2022). Stimulatory effects of extracellular vesicles derived from *Leuconostoc*
*holzapfelii* that exists in human scalp on hair growth in human follicle dermal papilla cells. Curr Issues Mol Biol.

[20] Kao MS, Wang Y, Marito S (2016). The mPEG-PCL copolymer for selective fermentation of *Staphylococcus*
*lugdunensis* against candida parapsilosis in the human microbiome. J Microb Biochem Technol.

[21] Vecino X, Rodríguez-López L, Ferreira D, Cruz JM, Moldes AB, Rodrigues LR (2018). Bioactivity of glycolipopeptide cell-bound biosurfactants against skin pathogens. Int J Biol Macromol.

[22] Tsai WH, Chou CH, Huang TY (2021). Heat-killed *Lactobacilli* preparations promote healing in the experimental cutaneous wounds. Cells.

[23] Tsai WH, Chou CH, Chiang YJ, Lin CG, Lee CH (2021). Regulatory effects of *Lactobacillus*
*plantarum*-GMNL6 on human skin health by improving skin microbiome. Int J Med Sci.

[24] Jhong JH, Tsai WH, Yang LC (2022). Heat-killed *Lacticaseibacillus*
*paracasei* GMNL-653 exerts antiosteoporotic effects by restoring the gut microbiota dysbiosis in ovariectomized mice. Front Nutr.

[25] Puebla-Barragan S, Reid G (2021). Probiotics in cosmetic and personal care products: trends and challenges. Molecules.

[26] Reygagne P, Bastien P, Couavoux MP (2017). The positive benefit of *Lacticaseibacillus*
*paracasei* NCC2461 ST11 in healthy volunteers with moderate to severe dandruff. Benef Microbes.

[27] Angiolella L, Leone C, Rojas F, Mussin J, de Los Angeles Sosa M, Giusiano G (2018). Biofilm, adherence, and hydrophobicity as virulence factors in *Malassezia*
*furfur*. Med Mycol.

[28] Vlachos C, Henning MAS, Gaitanis G, Faergemann J, Saunte DM (2020). Critical synthesis of available data in *Malassezia*
*folliculitis* and a systematic review of treatments. J Eur Acad Dermatol Venereol.

[29] Hamdino M, Saudy AA, El-Shahed LH, Taha M (2022). Identification of Malassezia species isolated from some Malassezia associated skin diseases. J Mycol Med.

[30] Prohic A, Simic D, Sadikovic TJ, Krupalija-Fazlic M (2014). Distribution of Malassezia species on healthy human skin in Bosnia and Herzegovina: correlation with body part, age and gender. Iran J Microbiol.

[31] Honnavar P, Chakrabarti A, Dhaliwal M, Dogra S, Handa S, Lakshmi PVM, Rudramurthy SM (2021). Sociodemographic characteristics and spectrum of Malassezia species in individuals with and without seborrhoeic dermatitis/dandruff: a comparison of residents of the urban and rural populations. Med Mycol.

[32] Hiruma M, Cho O, Hiruma M, Kurakado S, Sugita T, Ikeda S (2014). Genotype analyses of human commensal scalp fungi, *Malassezia*
*globosa*, and *Malassezia*
*restricta* on the scalps of patients with dandruff and healthy subjects. Mycopathologia.

[33] Zhang H, Ran Y, Xie Z, Zhang R (2013). Identification of *Malassezia* species in patients with seborrheic dermatitis in China. Mycopathologia.

[34] James AG, Abraham KH, Cox DS, Moore AE, Pople JE (2013). Metabolic analysis of the cutaneous fungi *Malassezia*
*globosa* and *M.*
*restricta* for insights on scalp condition and dandruff. Int J Cosmet Sci.

[35] Meray Y, Gençalp D, Güran M (2018). Putting it all together to understand the role of *Malassezia*
*spp.* in dandruff etiology. Mycopathologia.

[36] Clavaud C, Jourdain R, Bar-Hen A, Tichit M, Bouchier C, Pouradier F, Rawadi CE, Guillot J, Menard-Szczebara F, Breton L (2013). Dandruff is associated with disequilibrium in the proportion of the major bacterial and fungal populations colonizing the scalp. PLoS One.

[37] Wang L, Clavaud C, Bar-Hen A, Cui M, Gao J, Liu Y, Liu C, Shibagaki N, Gueniche A, Jourdain R (2015). Characterization of the major bacterial-fungal populations colonizing dandruff scalps in Shanghai, China, shows microbial disequilibrium. Exp Dermatol.

[38] Park T, Kim HJ, Myeong NR, Lee HG, Kwack I, Lee J, Kim BJ, Sul WJ, An S (2017). Collapse of human scalp microbiome network in dandruff and seborrhoeic dermatitis. Exp Dermatol.

[39] Sommer B, Overy DP, Kerr RG (2015). Identification and characterization of lipases from *Malassezia*
*restricta*, a causative agent of dandruff. FEMS Yeast Res.

[40] Park M, Lee JS, Jung WH, Lee YW (2020). pH-dependent expression, stability, and activity of *Malassezia*
*restricta* MrLip5 Lipase. Ann Dermatol.

[41] Adalsteinsson JA, Kaushik S, Muzumdar S, Guttman-Yassky E, Ungar J (2020). An update on the microbiology, immunology and genetics of seborrheic dermatitis. Exp Dermatol.

[42] Dawson TL (2007). Malassezia globosa and restricta: breakthrough understanding of the etiology and treatment of dandruff and seborrheic dermatitis through whole-genome analysis. J Investig Dermatol Symp Proc.

[43] Honnavar P (2017). β-Endorphin enhances the phospholipase activity of the dandruff causing fungi *Malassezia*
*globosa* and *Malassezia*
*restricta*. Med Mycol.

[44] Pinto D, Sorbellini E, Marzani B, Rucco M, Giuliani G, Rinaldi F (2019). Scalp bacterial shift in alopecia areata. PLoS One.

[45] Wang E, Lee JS, Hee TH (2012). Is *Propionibacterium*
*acnes* associated with hair casts and alopecia?. Int J Trichology.

[46] Ho BS, Ho EXP, Chu CW (2019). Microbiome in the hair follicle of androgenetic alopecia patients. PLoS One.

[47] Filaire E, Dreux A, Boutot C, Ranouille E, Berthon JY (2020). Characteristics of healthy and androgenetic alopecia scalp microbiome: effect of Lindera strychnifolia roots extract as a natural solution for its modulation. Int J Cosmet Sci.

[48] Bay L, Ring HC. Human skin microbiota in health and disease: the cutaneous communities’ interplay in equilibrium and dysbiosis: the cutaneous communities’ interplay in equilibrium and dysbiosis. APMIS. 2021;130:706–18. 10.1111/apm.13201 34919288

[49] Carvalho MJ, S Oliveira AL, Santos Pedrosa S, Pintado M, Pinto-Ribeiro I, Madureira AR. Skin microbiota and the cosmetic industry. Microb Ecol. 2022. 10.1007/s00248-022-02070-0. 10.1007/s00248-022-02070-0 35809121

[50] Meloni M, Balzaretti S, Collard N, Desaint S, Laperdrix C (2021). Reproducing the scalp microbiota community: co-colonization of a 3D reconstructed human epidermis with *C.*
*acnes* and *M.*
*restricta*. Int J Cosmet Sci.

[51] Kang SS, Sim JR, Yun CH, Han SH (2016). Lipoteichoic acids as a major virulence factor causing inflammatory responses via Toll-like receptor 2. Arch Pharm Res.

[52] Lee D, Im J, Park DH, Jeong S, Park M, Yoon S, Park J, Han SH (2021). *Lactobacillus*
*plantarum* Lipoteichoic acids possess strain-specific regulatory effects on the biofilm formation of dental pathogenic bacteria. Front Microbiol.

[53] Stenn KS, Paus R (2001). Controls of hair follicle cycling. Physiol Rev.

[54] Fukao M, Zendo T, Inoue T (2019). Plasmid-encoded glycosyltransferase operon is responsible for exopolysaccharide production, cell aggregation, and bile resistance in a probiotic strain, *Lactobacillus*
*brevis* KB290. J Biosci Bioeng.

