# Supplementary material for: Heat-killed Lacticaseibacillus paracasei GMNL-653 ameliorates human scalp health by regulating scalp microbiome
Source: BMC Microbiol. 2023 Apr 29;23:121. doi: 10.1186/s12866-023-02870-5 (PMC10148562; doi:10.1186/s12866-023-02870-5)
Supplement: Supplementary file 1 — Additional file 1: Table S1. The primer sequences of hair follicle growth factor-related genes (A) and hair follicle microbiota-related genes (B). Table S2. Correlations of the fungus or the bacteria with scalp conditions in the shampoo clinical trial. Figure S1. Heat-killed L. paracasei GMNL-653 modulates microbiota diversity in the subgroups of normal dandruff (A), high dandruff (B), normal oil (C), high oil (D), normal hair (E), and less hair (F). Start group (Start) described as the scalp in the beginning without any shampoo treatment. Control group (Ctrl) represented as the use of control shampoo without adding heat-killed GMNL-653 for 1 month. GMNL-653 group (GMNL-653) meant the use of heat-killed GMNL-653 shampoo for 1 month after using Ctrl shampoo. * p < 0.05 compared with Start group. # p < 0.05 compared with the Ctrl group. Figure S2. Hair volume is positive-correlated with accumulation of L. paracasei in human scalp after using heat-killed GMNL-653 shampoo for 2 months (A) and 4 months (B). Pearson correlation coefficient (R) and p value were calculated by SPSS statistics. [file 12866_2023_2870_MOESM1_ESM.pdf]

Supplementary Information

Table S1. The primer sequences of hair follicle growth factor-related genes (**A**) and hair follicle microbiota-related genes (**B**)

**A. human hair follicle growth factor related genes**		
**Gene name**	**Forward (5' to 3')**	**Reverse (5' to 3')**
ACTB	CCTTGGCATCCACGAAACT	TCTCCTTCTGCATCCTGTCG
IGF-1	TGCTCTCAACATCTCCCATC	GAAGAGATGCGAGGAGGACA
IGF-1R	GGCATACCTCAACGCCAATA	CAGCCCTTTCCCTCCTTT
VEGF	GCACCCATGGCAGAAGG	CTCGATTGGATGGCAGTAGCT
KGF	CCTGAGCGACACACAAGAAG	CACAATTCCAACTGCCACTG
**B. hair follicle microbiota related genes**		
**Gene name**	**Forward (5' to 3')**	**Reverse (5' to 3')**
Total bacteria	GTGSTGCAYGGYTGTCGTCA	ACGTCRTCCMCACCTTCCTC
L. paracasei	CAGACACAGATCAGCTTG	AACTTGGCATCCTTCAAA
S. epidermidis	GGCAAATTTGTGGGTCAAGA	TGGCTAATGGTTTGTCACCA
C. acnes	GCGTGAGTGGACGGTAATGGGTA	TTCCGACGCGATCAACCA
Malassezia	CTAAATATCGGGGAGAGACCGA	GTACTTTTAACTCTCTTTCCAAAGTGCTT
M. restricta	GTGAATTGCAGAATTCCGTGAAT	GCGAGCCTGTGCTAGGTA
M. globosa	GGCCAAGCGCGCTCT	CCACCACCAAATGCTCTCCTACAG

Table S2. Correlations of the fungus or the bacteria with scalp conditions in the shampoo clinical trial.

Correlation between		Sebum secretion	Hair growth (3M)	Hair growth (5M)	Dandruff
C. acnes	Pearson's r	0.333	-0.092	-0.07	0.01
	P value	0.009**	0.454	0.595	0.936
S. epidermidis	Pearson's r	0.048	-0.139	-0.145	0.278
	P value	0.715	0.277	0.269	0.024*
M. restricta	Pearson's r	-0.141	-0.113	-0.121	-0.017
	P value	0.288	0.382	0.359	0.891
M. globosa	Pearson's r	-0.133	0.107	0.027	0.026
	P value	0.315	0.408	0.839	0.837

* means $P < 0.05$ and ** means $P < 0.01$

3M and 5M: the timing of the 3rd and 5th month in Fig.4.

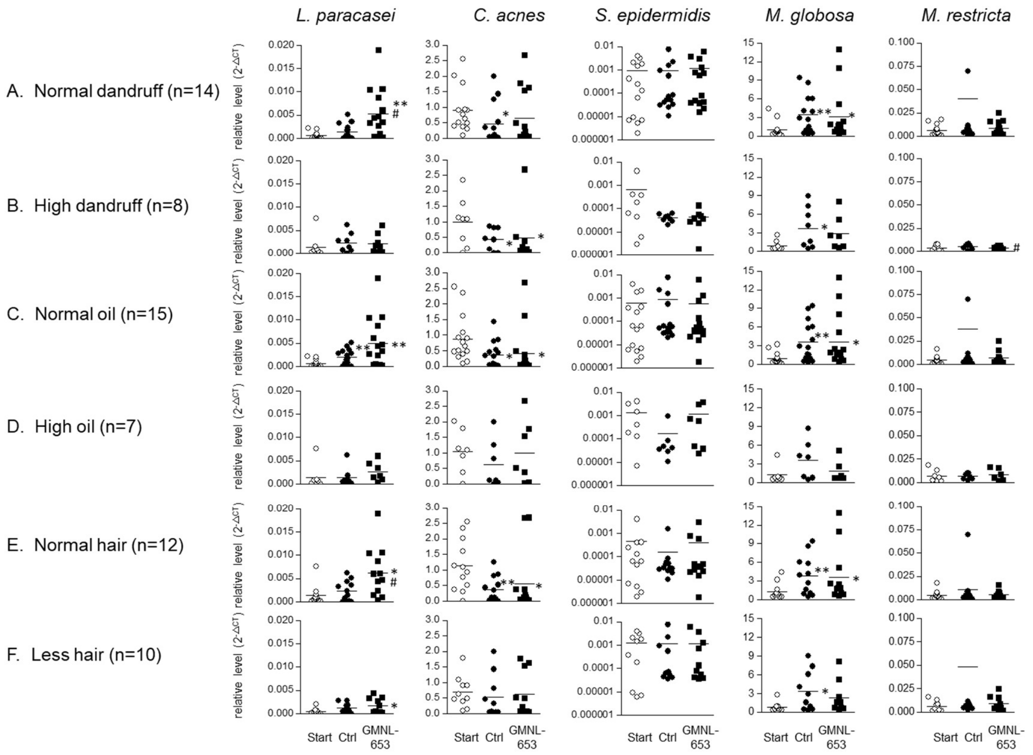

Figure S1. Heat-killed *L. paracasei* GMNL-653 modulates microbiota diversity in the subgroups of normal dandruff (**A**), high dandruff (**B**), normal oil (**C**), high oil (**D**), normal hair (**E**), and less hair (**F**). Start group (Start) described as the scalp in the beginning without any shampoo treatment. Control group (Ctrl) represented as the use of control shampoo without adding heat-killed GMNL-653 for 1 month. GMNL-653 group (GMNL-653) meant the use of heat-killed GMNL-653 shampoo for 1 month after using Ctrl shampoo. * $p < 0.05$ compared with Start group. # $p < 0.05$ compared with the Ctrl group.

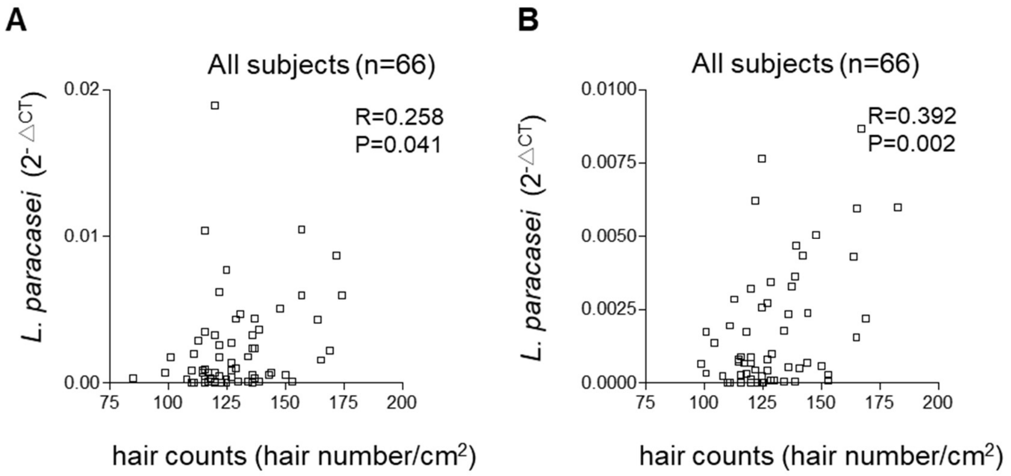

Figure S2. Hair volume is positive-correlated with accumulation of *L. paracasei* in human scalp after using heat-killed GMNL-653 shampoo for 2 months (**A**) and 4 months (**B**). Pearson correlation coefficient (R) and p value were calculated by SPSS statistics.
